# Hydroxyurea: A useful adjunct to the standard antiviral therapy in chronic hepatitis B

**Published:** 2011-03-01

**Authors:** Mostafa Alavi-Moghaddam

**Affiliations:** 1 Research Center for Gastroenterology and Liver Disease, Shahid Beheshti University of Medical Sciences, Tehran, IR Iran

**Keywords:** Hydroxyurea, chronic hepatitis B

Dear Editor,

I read with interest the article by Kashani et al, on the use of hydroxyurea (HU) in the treatment of chronic hepatitis B published in Hepatitis Monthly [1]. In their article, the authors performed a limited pilot study and concluded that HU effectively blocks HBV replication. Favorable dosing schedule, safety profile, and cost make HU a very attractive adjunct to the therapeutic armamentarium in a wide range of diseases including sickle cell anemia, thalassemia intermedia and human immunodeficiency virus (HIV) infection [2][3]. The potential role of HU in the treatment of HIV infection was supported first by in vitro experiments and then by controlled clinical trials [4]. Indeed, an activated cellular environment is crucial for the initial phase of the HIV-1 life cycle that involves reverse transcription of the viral RNA, the establishment of the HIV-1 provirus within host chromosomes, and the subsequent production of virions. The arrest of the cell cycle in the G1 phase by HU reduces cellular activation and thus should decrease viral production and overall viral burden [5]. In all controlled trials assessing treatment of HIV, addition of HU to the standard antiretroviral therapy (ART) has been compared to ART with or without placebo [4].Herein, I would like to mention a couple of drawbacks in the study design of this article: First of all, for ethical concerns, it was better if the authors would have used HU or placebo with standard antiviral therapy among patients and control groups, respectively, instead of first treating naïve chronic hepatitis B patients with HU. Furthermore, in this study, only results of HBV DNA viral load titers in cases are given and no information regarding HBe Ag status and liver enzyme profiles of the patients which have independent effects on standard antiviral treatment outcome, are provided. Finally, it is not clear if all samples for the HBV DNA viral loads were tested in one laboratory using a similar and standard method. There is also not clear whether assessors were blinded to the samples. Of course, causal inference for the efficacy of HU in the treatment of chronic hepatitis B patients should be accomplished through conduction of well-designed randomized controlled trials. Nonetheless, pilot studies conducted in this field should have enough power to justify conduction of future studies.
